# [^18^F]fallypride-PET/CT Analysis of the Dopamine D_2_/D_3_ Receptor in the Hemiparkinsonian Rat Brain Following Intrastriatal Botulinum Neurotoxin A Injection

**DOI:** 10.3390/molecules23030587

**Published:** 2018-03-06

**Authors:** Teresa Mann, Jens Kurth, Alexander Hawlitschka, Jan Stenzel, Tobias Lindner, Stefan Polei, Alexander Hohn, Bernd J. Krause, Andreas Wree

**Affiliations:** 1 Institute of Anatomy, Rostock University Medical Center, Gertrudenstrasse 9, 18057 Rostock, Germany; alexander.hawlitschka@med.uni-rostock.de (A.H.); andreas.wree@med.uni-rostock.de (A.W.); 2 Department of Nuclear Medicine, Rostock University Medical Centre, Gertrudenplatz 1, 18057 Rostock, Germany; jens.kurth@med.uni-rostock.de (J.K.); alexander.hohn@med.uni-rostock.de (A.H.); bernd.krause@med.uni-rostock.de (B.J.K.); 3 Core Facility Multimodal Small Animal Imaging, Rostock University Medical Center, Schillingallee 69a, 18057 Rostock, Germany; Jan.Stenzel@med.uni-rostock.de (J.S.); tobias.lindner@med.uni-rostock.de (T.L.); stefan.polei@gmx.de (S.P.)

**Keywords:** D_2_/D_3_ receptors, hemiparkinsonian rat model, Botulinum neurotoxin A, basal ganglia, striatum, Parkinson’s disease, small animal imaging, PET/CT, [^18^F]fallypride, MRI

## Abstract

Intrastriatal injection of botulinum neurotoxin A (BoNT-A) results in improved motor behavior of hemiparkinsonian (hemi-PD) rats, an animal model for Parkinson’s disease. The caudate–putamen (CPu), as the main input nucleus of the basal ganglia loop, is fundamentally involved in motor function and directly interacts with the dopaminergic system. To determine receptor-mediated explanations for the BoNT-A effect, we analyzed the dopamine D_2_/D_3_ receptor (D_2_/D_3_R) in the CPu of 6-hydroxydopamine (6-OHDA)-induced hemi-PD rats by [^18^F]fallypride-PET/CT scans one, three, and six months post-BoNT-A or -sham-BoNT-A injection. Male Wistar rats were assigned to three different groups: controls, sham-injected hemi-PD rats, and BoNT-A-injected hemi-PD rats. Disease-specific motor impairment was verified by apomorphine and amphetamine rotation testing. Animal-specific magnetic resonance imaging was performed for co-registration and anatomical reference. PET quantification was achieved using PMOD software with the simplified reference tissue model 2. Hemi-PD rats exhibited a constant increase of 23% in D_2_/D_3_R availability in the CPu, which was almost normalized by intrastriatal application of BoNT-A. Importantly, the BoNT-A effect on striatal D_2_/D_3_R significantly correlated with behavioral results in the apomorphine rotation test. Our results suggest a therapeutic effect of BoNT-A on the impaired motor behavior of hemi-PD rats by reducing interhemispheric changes of striatal D_2_/D_3_R.

## 1. Introduction

Positron emission tomography (PET) using the radioligand [^18^F]fallypride enables in vivo detection of disease-specific alterations of the dopaminergic system, more precisely of D_2_/D_3_ receptor (D_2_/D_3_R) availability. Small animal PET hybrid tomographs allow imaging and quantification of D_2_/D_3_R binding by the use of [^18^F]fallypride in rodent models of neurodegenerative disorders like Parkinson’s disease (PD) [1,2,3]. [^18^F]fallypride is characterized by high sensitivity and selectivity for D_2_R; for instance, administration of the D_2_R antagonist haloperidol blocked specific [^18^F]fallypride binding in the mouse caudate–putamen (CPu) by 95% [4,5]. Besides specific binding to D_2_R, [^18^F]fallypride displays affinity to the D_2_-like D_3_R, about 20% of the radioligand bind to D_3_R in vivo in small animals [6]. In PD a drastic loss of striatal dopamine (DA) caused by progressing degeneration of dopaminergic neurons in the substantia nigra pars compacta (SNpc) results in imbalanced neurotransmitter systems and underlies motor complications. Moreover, due to DA depletion and the subsequent missing inhibition of striatal cholinergic interneurons, hypercholinism is an additional feature of PD exacerbating motor impairment [7,8]. Interestingly, there are physiological bidirectional regulating effects of the cholinergic and dopaminergic system [9] via complex involvement of muscarinic and nicotinic receptors on DA release from dopaminergic terminals [10,11].

For causal analysis and development of novel therapeutics for PD in preclinical research the experimental hemiparkinsonian (hemi-PD) is an accepted animal model [12]. Unilateral injection of 6-hydroxdopamine (6-OHDA) into the medial forebrain bundle (MFB) of rats provokes rapid dopaminergic depletion by auto-oxidation and consequent oxidative stress [13]. The near-complete loss of dopaminergic neurons in the SNpc via retrograde axonal transport of the toxin 6-OHDA after stereotaxic injection into the MFB mimics a late stage of PD [14]. Current therapeutic strategies for PD focus primarily on compensation of DA in the striatum (caudate–putamen, CPu) by either DA precursors [15,16] or DA receptor agonists [17]. Though, clinical efficiency is limited and chronic administrations leads to severe side effects like motor fluctuations and dyskinesia [18,19]. Other therapeutic options target the cholinergic system mostly by blocking muscarinic receptors or by inhibition of cholinesterase [20,21]. Systemic administration of anticholinergic substances causes severe side effects like confusion, dry mouth, blurred vision, and cognitive impairment [22].

Recently, we demonstrated that local injection of the anticholinergic Botulinum neurotoxin A (BoNT-A) significantly improved D_2_R agonist-induced asymmetric rotational behavior in hemi-PD rats [23,24,25,26]. BoNT-A acts mainly on cholinergic neurons and inhibits distribution of acetylcholine into the synaptic cleft via cleavage of the synaptosomal-associated protein of 25-kDa (SNAP25) [27,28,29]. The intracerebral injection of BoNT-A avoids severe side effects in both the central and peripheral nervous system [23]. Notably, intrastriatal application of BoNT-A does not cause cytotoxicity [30] or impaired cognition [24] in rats. As known from other medical implementation, BoNT-A demonstrates a transient therapeutic effect in hemi-PD rats that lasts up to six months post-injection [23,31]. To examine the longitudinal cellular mechanisms of the positive BoNT-A effect on receptor level, we performed [^18^F]fallypride-PET/CT scans one, three, and six months post-BoNT-A or -sham-BoNT-A injection and quantified D_2_/D_3_R availability in controls, sham-injected hemi-PD rats, and BoNT-A-injected hemi-PD rats. 

## 2. Results

D_2_/D_3_R availability was analyzed longitudinally in controls (sham-6-OHDA + sham-BoNT-A, *n* = 9), sham-injected hemi-PD rats (6-OHDA + sham-BoNT-A, *n* = 7) and BoNT-A-injected hemi-PD rats (6-OHDA + BoNT-A, *n* = 10) by dynamic [^18^F]fallypride-PET/CT scans.

### 2.1. Immunohistochemistry and Behavioral Testing

**Figure 1:**
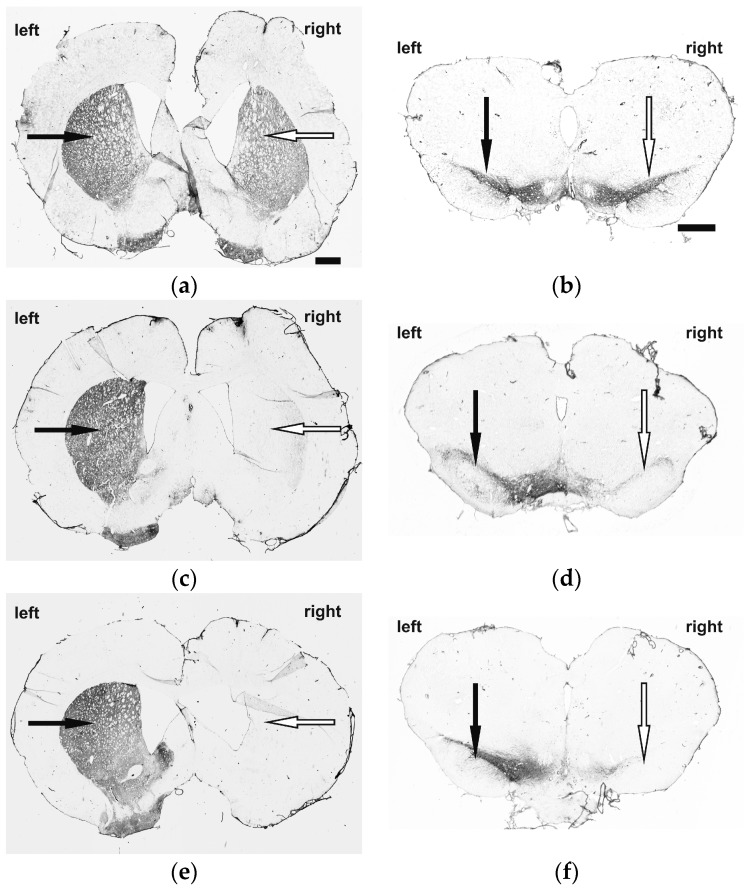
TH-immunoreactivity in the telencephalon (left column) and the mesencephalon (right column) of (**a**,**b**) controls (sham-6-OHDA + sham-BoNT-A) (**c**,**d**) sham-injected hemi-PD rats (6-OHDA + sham-BoNT-A) and (**e**,**f**) BoNT-A-injected hemi-PD rats (6-OHDA + BoNT-A). 6-OHDA or sham-6-OHDA was unilaterally injected into the MFB of the right hemisphere and BoNT-A was injected ipsilateral at two sites into the CPu. Controls showed symmetric TH pattern in the CPu (**a**,**c**,**e**; black (**left**) and white (**right**) arrow) and SN (**b**,**d**,**f**; black (**left**) and white (**right**) arrow), sham-injected hemi-PD rats demonstrated an almost complete loss of TH-positive cells in the CPu and SN and BoNT-A injection did not influence these findings in hemi-PD rats. The scale bar applies for **a**–**f** = 1 mm.

To qualitatively verify successful 6-OHDA-induced dopaminergic deafferentation we performed tyrosine hydroxylase (TH) immunostaining (for dopaminergic neurons). TH-reaction in the left and right CPu and SN of control rats (sham-6-OHDA + sham-BoNT-A) showed no loss of TH-reaction (Figure 1a,b). In hemi-PD rats (6-OHDA + sham-BoNT-A) an ipsilateral loss of almost all TH-immunoreactivity was visible in the CPu and SN, indicating dopaminergic deafferentation in the CPu due to dopaminergic cell loss in the SN (Figure 1c,d), and BoNT-A injection in hemi-PD rats (6-OHDA + BoNT-A) did not demonstrate an additive effect on these reaction patterns (Figure 1e,f). 

**Figure 2:**
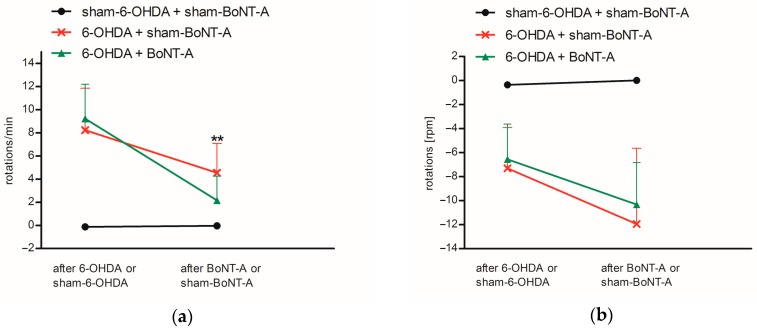
Results of the rotational behavior in (**a**) apomorphine- and (**b**) amphetamine-induced testing for controls (sham-6-OHDA + sham-BoNT-A), sham-injected hemi-PD rats (6-OHDA + sham-BoNT-A) and BoNT-A-injected hemi-PD rats (6-OHDA + BoNT-A) displayed after 6-OHDA or sham-6-OHDA and BoNT-A or sham-BoNT-A injection. Rotations contralateral to the injection side (clockwise) are displayed with negative algebraic signs, anti-clockwise rotations with positive algebraic signs. Controls did not demonstrate designated rotational behavior, hemi-PD rats exhibited strong asymmetric drug-induced rotations that were almost completely abolished after BoNT-A injection in apomorphine testing and slightly abolished in amphetamine testing. Significance is displayed as ** *p* < 0.01.

Asymmetric rotations of hemi-PD rats were tested using apomorphine- and amphetamine-induced rotations one month after 6-OHDA lesion. Also, the positive effect of intrastriatally injected BoNT-A on drug-induced rotations was analyzed two weeks after administration. All hemi-PD rats exhibited distinct apomorphine-induced rotations contralateral to the 6-OHDA lesion before BoNT-A or sham-BoNT-A injection of 8.2 ± 3.6 rpm (6-OHDA + sham-BoNT-A) and 9.2 ± 3.0 rpm (6-OHDA + BoNT-A), controls did not show rotational behavior (sham-6-OHDA + sham-BoNT-A) (Figure 2a). Sham injection in hemi-PD rats slightly decreased rotations to 4.6 ± 2.5 rpm (6-OHDA + sham-BoNT-A) and did not affect the behavior of controls (sham-6-OHDA + sham-BoNT-A). Following BoNT-A injection rotational behavior was reversed in hemi-PD rats to 2.2 ± 2.1 rpm (6-OHDA + BoNT-A). The positive motor effect of BoNT-A in hemi-PD rats was significant compared to sham injections (*p* = 0.021) (Figure 2a). Before BoNT-A or sham-BoNT-A injection amphetamine administration caused ipsilateral rotations in hemi-PD rats of −7.3 ± 3.4 rpm (6-OHDA + sham-BoNT-A) and −6.6 ± 2.9 rpm (6-OHDA + BoNT-A) but not in controls (sham-6-OHDA + sham-BoNT-A) (Figure 2b). After BoNT-A or sham-BoNT-A injection asymmetric rotational behavior was with −12.0 ± 6.3 rpm (6-OHDA + BoNT-A) and −10.3 ± 3.5 rpm (6-OHDA + sham-BoNT-A) further increased and BoNT-A effect was compared to sham injection not significantly abolished. Sham-BoNT-A injection in controls (sham-6-OHDA + sham-BoNT-A) did not result in ipsilateral rotations (Figure 2b).

### 2.2. Striatal D_2_/D_3_R Availability

**Figure 3:**
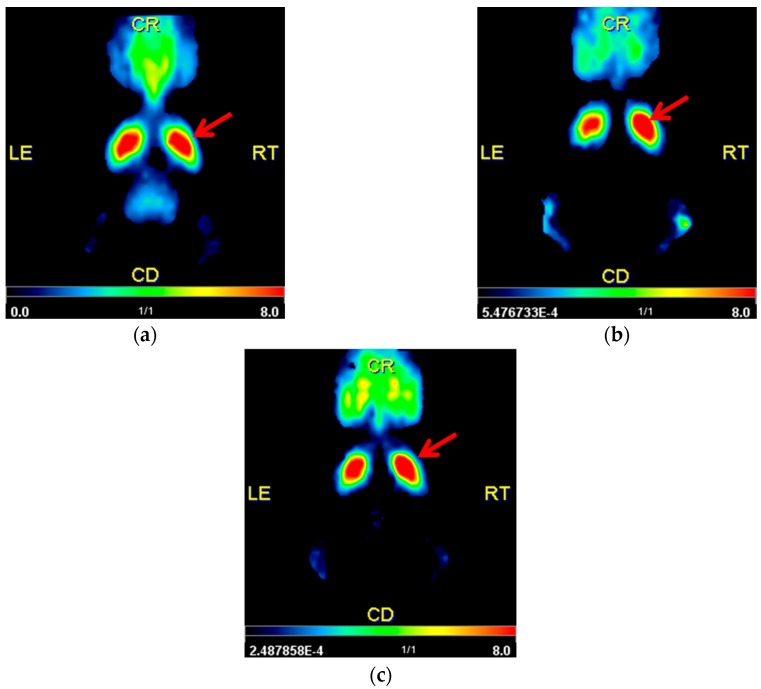
Qualitative analysis using pixel-wised parametric mapping for the parameter BP_nd_ showing the left and right CPu in transversal sections for (**a**) controls (sham-6-OHDA + sham-BoNT-A) (**b**) sham-injected hemi-PD rats (6-OHDA + sham-BoNT-A) and (**c**) BoNT-A-injected hemi-PD rats (6-OHDA + BoNT-A) one month after BoNT-A or sham-BoNT-A injection. A representative animal of each experimental group was used. Controls did not reveal visual side differences, an increased signal of BP_nd_ distribution in the right CPu of hemi-PD rats was clearly visible and BoNT-A injection normalized the increased signal in hemi-PD rats. The right CPu is marked with a red arrow.

Qualitative analysis with parametric mapping of non-displaceable binding potential (BP_nd_) revealed no obvious interhemispheric differences for controls (sham-6-OHDA + sham-BoNT-A). However, increased signals in the right CPu of hemi-PD rats were visible (6-OHDA + sham-BoNT-A) compared to the unaffected side. This visual right–left difference was diminished after BoNT-A injection into the right CPu of hemi-PD rats (6-OHDA + BoNT-A) (Figure 3a–c). 

**Figure 4:**
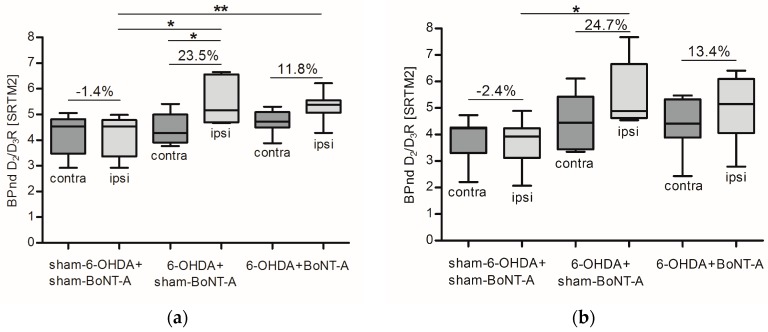

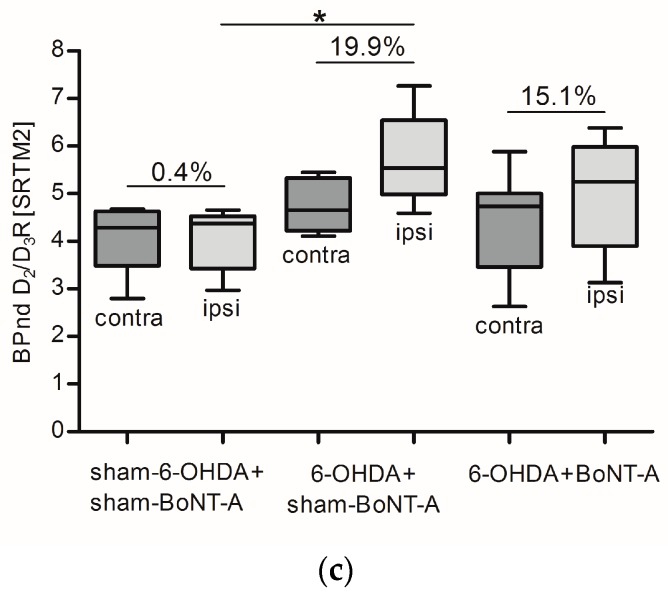
Box plots for BP_nd_ values of D_2_/D_3_R depicting median and interquartile ranges separately for the contralateral (dark grey) and ipsilateral (light grey) CPu for controls (sham-6-OHDA + sham-BoNT-A), sham-injected hemi-PD rats (6-OHDA + sham-BoNT-A) and BoNT-A-injected hemi-PD rats (6-OHDA + BoNT-A) (**a**) one month, (**b**) three months, and (**c**) six months post-BoNT-A or -sham-BoNT-A injection. D_2_/D_3_R availability was consistently symmetric in controls, increased in sham-injected hemi-PD rats at all analyzed time points and was reduced to nearly normal values after BoNT-A injection in hemi-PD rats. Significance is displayed as * *p* < 0.05, ** *p* < 0.01.

For quantification the simplified reference tissue model 2 (SRTM2) was applied and BP_nd_ was estimated separately for the left and right striatum. Controls (sham-6-OHDA + sham-BoNT-A) revealed no relative interhemispheric right–left differences: mean BP_nd_ of 4.2 ± 0.8/4.2 ± 0.8 (left/right CPu) one month post-sham-BoNT-A injection, 3.8 ± 0.8/3.7 ± 0.9 (left/right CPu) three months post-sham-BoNT-A injection and 4.1 ± 0.7/4.1 ± 0.7 (left/right CPu) six months post-sham-BoNT-A injection were found (Figure 4a,c; Table 1). Hemi-PD rats that received sham-BoNT-A injection (6-OHDA + sham-BoNT-A) exhibited strong interhemispheric right–left differences of about 23%: kinetic analysis resulted in mean BP_nd_ values of 4.4 ± 0.6/5.5 ± 0.9 (left/right CPu) one month post-sham-BoNT-A injection, 4.4 ± 1.1/5.5 ± 1.3 (left/right CPu) three months post-sham-BoNT-A injection, and 4.8 ± 1.0/5.7 ± 1.0 (left/right CPu) six months post-sham-BoNT-A injection. The contralateral CPu was never affected and displayed very stable BP_nd_ throughout all experimental groups and scanning time points (Figure 4a–c; Table 1). The increase in BP_nd_ in the right CPu of hemi-PD rats (6-OHDA + sham-BoNT-A) was significant compared to the left CPu of the same experimental group one month post-sham-BoNT-A injection (*p* = 0.047) (Figure 4a) and compared to the right CPu of controls (sham-6-OHDA + sham-BoNT-A) at all 3 examined time points (*p* = 0.03, *p* = 0.064, *p* = 0.039) (Figure 4a–c). BoNT-A injection in hemi-PD rats (6-OHDA + BoNT-A) reduced relative interhemispheric right–left difference to about 13.4%: quantification revealed mean BP_nd_ of 4.7 ± 0.4/5.3 ± 0.5 (left/right CPu) one month post-BoNT-A injection, 4.4 ± 1.0/5.0 ± 1.2 (left/right CPu) three months post-BoNT-A injection and 4.4 ± 1.0/5.1 ± 1.1 (left/right CPu) six months post-BoNT-A injection (Figure 4a–c; Table 1). The BoNT-A effect was significant compared to the ipsilateral CPu of controls (sham-6-OHDA + sham-BoNT-A) one month post-BoNT-A injection (*p* = 0.0087) and showed a transient course throughout the timeline (Figure 4a–c). A list of all individual values for BP_nd_ expressing D_2_/D_3_R availability separately for the left and right CPu and the relative interhemispheric right–left difference in each of the 26 analyzed rats is displayed in Table 1.

### 2.3. Correlation of D_2_/D_3_R Side Differences and Behavior

**Figure 5:**
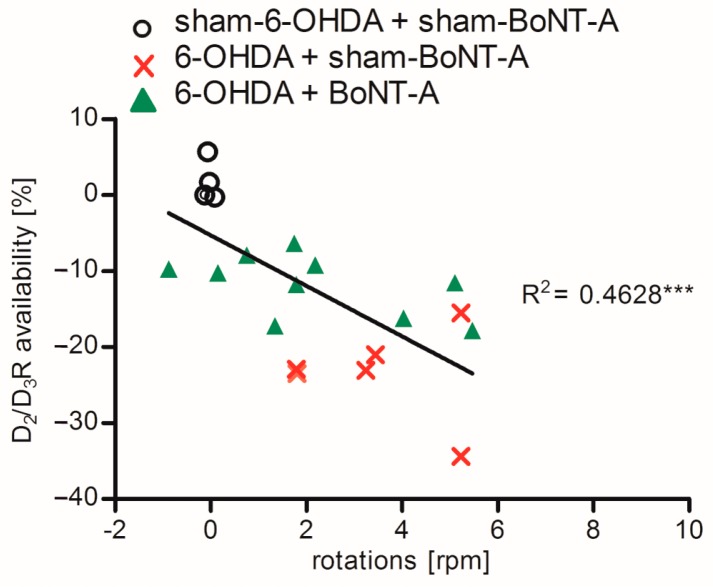
Linear correlation of right–left differences of D_2_/D_3_R availability in (%) and apomorphine-induced rotations one month after BoNT-A or sham-BoNT-A injection for controls (sham-6-OHDA + sham-BoNT-A), sham-injected hemi-PD rats (6-OHDA + sham-BoNT-A) and BoNT-A-injected hemi-PD rats (6-OHDA + BoNT-A). Controls did neither demonstrate interhemispheric differences nor rotational behavior. With increasing right–left differences also asymmetric rotations increased in hemi-PD rats and both was normalized after BoNT-A injection. Significance is displayed as *** *p* < 0.001.

A possible correlation of the degree of interhemispheric differences in D_2_/D_3_R availability and apomorphine-induced rotations for controls (sham-6-OHDA + sham-BoNT-A), sham-injected hemi-PD rats (6-OHDA + sham-BoNT-A) and BoNT-A-injected hemi-PD rats (6-OHDA + BoNT-A) was examined one month post-BoNT-A or -sham-BoNT-A injection. Controls (sham-6-OHDA + sham-BoNT-A) did not demonstrate right–left differences or rotational behavior. With increasing right–left differences contralateral rotations of hemi-PD rats (6-OHDA + sham-BoNT-A) increased and also the normalizing effect on interhemispheric D_2_/D_3_R differences after BoNT-A injection (6-OHDA + BoNT-A) was connected with behavior. A highly significant relationship between increasing right–left differences, expressing a higher D_2_/D_3_R availability in the right CPu, and the apomorphine-induced rotational behavior was found (*p* = 0.0007) (Figure 5). 

## 3. Discussion

In this study we examined cellular mechanisms of the positive motor effect of intrastriatally injected BoNT-A by [^18^F]fallypride PET/CT scans in hemi-PD rats, as BoNT-A was previously demonstrated to abolish apomorphine-induced rotational behavior in 6-OHDA-lesioned [23,24,25,26]. The control group respected the entire surgical procedure as the minimal lesion caused by the insertion of the syringe could lead to changes in receptor binding sites [32,33]. We did not include an experimental group studying BoNT-A in sham-lesioned rats in our design as we assumed that BoNT-A would not alter per se the expression of D_2_/D_3_ receptors. Indeed, we have previously performed extensive in vitro analysis of D_2_/D_3_ receptors as well as apomorphine-induced rotational behavior in BoNT-A-injected rats earlier and did not find major effects [31]. 

Hemi-PD rats demonstrated a constant contralateral rotational behavior after apomorphine injection and rather inconsistent amphetamine-induced rotations four weeks after 6-OHDA lesion (Figure 2a,b). A period of four weeks before behavioral testing was left to ensure maximum dopaminergic deafferentation, as dopaminergic cell death [34] as well as consequent plasticity effects [31] last up to four weeks after injection of 6-OHDA. Notably, increasing right–left differences of D_2_/D_3_R availability significantly correlated with increasing asymmetry in apomorphine-induced rotations (Figure 5). Rotational tests using the D_2_ agonist apomorphine or the DA releaser amphetamine are commonly used to detect the degree of dopaminergic deafferentation in hemi-PD rats. As apomorphine acts on increased striatal D_2_/D_3_R in the DA-depleted CPu of hemi-PD rats [35,36,37], it leads to a larger inhibition of the right CPu and as a consequence to an elevated motor urge to the contralateral side. Resulting rotations to the left of more than four rotations per minute confirm dopaminergic degeneration of more than 90% [25,38,39,40]. Amphetamine induces DA release form nerve terminals and strongly affects the non-lesioned hemisphere of hemi-PD rats, which begin to turn to the ipsilateral side of the 6-OHDA lesion [41]. In line with our findings, another study demonstrated that apomorphine but not amphetamine is a reliable indicator for maximal dopaminergic cell death in hemi-PD rats [42]. 

Dynamic [^18^F]fallypride PET/CT scans over 90 min revealed an increase of 23% in D_2_/D_3_R availability being consistent up to six months post-6-OHDA lesion and a normalization of this pathological imbalance after BoNT-A injection into the CPu of hemi-PD rats (Figure 3 and Figure 4; Table 1). Unlike [^11^C]raclopride, [^18^F]fallypride is not easily displaced by endogenous DA, as demonstrated in monkey [43], human [44] and rat brain [45]. To cover the transient effect of BoNT-A demonstrated previously [23], we performed longitudinal measurements using [^18^F]fallypride in the same rodent. This seemed feasible as repeated measurements with [^18^F]fallypride PET/CT exhibited only small variations in mice [5] and also in our study, controls (sham-6-OHDA + sham-BoNT-A) did not show variations in BP_nd_ comparing the three PET/CT scanning time points (Figure 3a and Figure 4a–c ; Table 1).

Our finding of a constant increase of about 23% in D_2_/D_3_R availability in hemi-PD rats is in line with a number of similar studies both in vitro and in vivo. Unilateral injection of 6-OHDA into the MFB or SNpc of rats resulted in a consistent increase of D_2_R density of 20% to 40% subject to the injected dosage and survival time analyzed using in vitro autoradiography [35,46,47,48,49,50]. Also, in vivo PET/CT analyses with [^11^C]raclopride and [^18^F]fallypride are in accordance with our results. [^11^C]raclopride PET demonstrated an ipsilateral increase of 17% to 27.7% [2] and approximately 35% [51] in D_2_R availability in hemi-PD rats after MFB injection and an increase of 23% [52] and 16.6% [53] after 6-OHDA lesion of the SNpc. [^18^F]fallypride PET/CT scans revealed an 12% increase in D_2_/D_3_R availability in hemi-PD rats after injection of 6-OHDA into the CPu [2].

Intrastriatally injections with BoNT-A significantly reduced the pathologically increased D_2_/D_3_R availability in hemi-PD rats (Figure 3c and Figure 4) and significantly abolished apomorphine-induced rotations (Figure 2a). Apomorphine-induced rotations were also moderately decreased after sham-BoNT-A injection in hemi-PD rats. This effect is likely to be caused by minimal mechanical damage caused by insertion of the cannula into the CPu and injection of sham solution. One might argue that the positive BoNT-A effect is caused by simple striatal cell death after BoNT-A injection, as D_2_/D_3_R are localized on medium spiny neurons (MSN), presynapses of cholinergic interneurons and boutons of dopaminergic afferents in the CPu [54,55,56]. Previously we demonstrated that BoNT-A injection did neither cause striatal neuronal loss or reduced volume [26] nor death of cholinergic interneurons in the CPu [30]. The positive BoNT-A effect diminished with increasing post-injection time. The BoNT-A effect on D_2_/D_3_R in hemi-PD rats has been analyzed before in a quantitative in vitro autoradiography study demonstrating a normalizing effect, which significantly correlates with apomorphine-induced rotations [31].

A preceding study performed [^11^C]raclopride PET to analyze the effect of BoNT-A on pathological increased D_2_/D_3_R and found a positive effect on pathological increased D_2_R availability in hemi-PD rats [3]. Here, we extended this experimental setup by introducing a control group (sham-6-OHDA + sham-BoNT-A) to investigate both ipsi- and contralateral effects of tissue damage by cannula injection and increased group size to substantiate possible significant effects. Moreover, we used the more specific radioligand [^18^F]fallypride instead of [^11^C]raclopride and conducted animal-specific MRI scans for co-registration with CT-corrected PET data to improve data analysis by making use of the high morphologic resolution of MRI.

Quantification was subsequently performed using SRTM2 [57] for kinetic modeling, having the advantage of no need for arterial blood sampling and being established especially for neuroimaging studies [58]. Application of kinetic models allows quantitative determination of transfer rates and provides in depth understanding of physiological parameters. The cerebellum was used as the reference region being devoid of D_2_/D_3_R [59] and being validated as a suitable reference region for D_2_/D_3_R before [60,61,62]. As spatial resolution of PET scans is a limiting factor especially in small animals [63], we used animal-specific MRI scans for spatial normalization, thus high precision of Voxels of interest (VOI) delineation and almost full recovery could be guaranteed in our study. Partial volume effects (PVE) occur mostly in structures being smaller than about 2 times of the width at half maximum of the scanner [64,65]. As our rats exhibited striatal volumes of about 35 to 50 mm^2^ [26,66] and the utilized scanner is characterized by a spatial resolution of 1.5 mm, the CPu as the target region exceeded the critical size for the occurrence of PVE. Moreover, the CPu seems to be only minimally effected by extracranial gland and skull activity resulting from its deep localization within the brain [6]. Due to predominantly relative comparisons between hemispheres within treatment groups in our study setup, a potentially emerging PVE would be mathematically shortened. Altogether, in this study we decided to omit PVE correction for quantification of [^18^F]fallypride uptake in the CPu of hemi-PD rats. Also in comparable studies, no correction of the PVE seemed necessary for quantification of radioligands demonstrating very specific and region-limited binding kinetics like [^18^F]fallypride [2] or [^11^C]raclopride [67]. Nevertheless, a methodical consideration of this aspect might be of high interest for further investigations. Another critical point of small animal imaging is the need for anesthesia, commonly realized by respiration of isoflurane. For analyzing the availability of D_2_/D_3_R effects of isoflurane seem not pivotal, as the BP of D_2_R measured with [^11^C]raclopride was only marginally changed in mice [5] and [^18^F]fallypride uptake did not differ in awake rats that received late isoflurane anesthesia compared to rats under continuous anesthesia [61].

## 4. Materials and Methods

### 4.1. Animals

**Figure 6:**
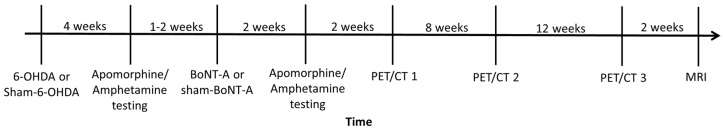
Timeline of the study design. Hemi-PD was unilaterally induced by 6-OHDA injection into the right MFB. Controls received sham-6-OHDA injection. The degree of dopaminergic cell loss was verified with apomorphine- and amphetamine-induced behavioral testing. Five to six weeks after the 6-OHDA or sham-6-OHDA injection, rats obtained BoNT-A or sham-BoNT-A injection into the ipsilateral CPu. The positive effect on the motor behavior of BoNT-A was then controlled in rotation tests. Subsequently, each rat was scanned by [^18^F]fallypride-PET/CT analysis one, three and six months post-BoNT-A or -sham-BoNT-A injection. A final MRI scan was performed as anatomical reference for PET/CT imaging.

Twenty-six male Wistar rats (Charles River WIGA, Sulzfeld, Germany; RRID: RGD_737929) either assigned to controls (sham-6-OHDA + sham-BoNT-A, *n* = 9), sham-injected hemi-PD rats (6-OHDA + sham-BoNT-A, *n* = 7) or BoNT-A-injected hemi-PD rats (6-OHDA + BoNT-A, *n* = 10) were included in the experiments. Housing was conducted under standard conditions (22 ± 2 °C, 12 h day-and-night cycle) in a fully air-conditioned room with access to water and food ad libitum. The research protocol and all experimental procedures fulfilled legal obligations of the animal welfare act and were approved by the state Animal Research Committee of Mecklenburg–Western Pomerania (LALLF M-V/7221.3-1-005/16, approval: 03/08/2016). The timeline of the experimental setup is presented in Figure 6.

### 4.2. 6-OHDA and BoNT-A Injection

Animals received 6-OHDA or sham-6-OHDA injection at a weight of 285–305 g in a stereotaxic operation and BoNT-A or sham-BoNT-A injection five to six weeks later. Anesthesia was induced with a ketamine-xylazine mixture (ketamine: 50 mg/kg, xylazine: 4 mg/kg). Dopaminergic cell death was provoked by unilateral injection of 24 μg of 6-OHDA (Sigma-Aldrich, St. Louis, MO, USA) dissolved in 4 μL 0.1 M citrate buffer into the right MFB. Sham-6-OHDA animals received only citrate buffer. Exact coordinates of 6-OHDA or sham-6-OHDA injection were anterior-posterior = −2.3 mm, lateral = −1.5 mm and ventral = −9.0 mm [68]. BoNT-A (Lot#13029A1; List, Campbell, CA, USA; purchased via Quadratech, Epsom, UK) supplemented with phosphate-buffered saline (PBS) and 0.1% bovine serum albumin (BSA) was injected at two sites into the right CPu (total dose: 1 ng). Sham-BoNT-A animals received only PBS + BSA. Exact coordinates of BoNT-A or sham-BoNT-A injection were anterior-posterior = +1.3 mm/−0.4 mm, lateral = −2.6 mm/−3.6 mm and ventral = −5.5 mm/−5.5 mm [68]. For application of either 6-OHDA, BoNT-A or sham solution a 5 μL Hamilton syringe was used and the respective volume was continuously delivered over a time span of 4 min. Afterwards, the needle was left in place for another 5 min to avoid reflux. 

### 4.3. Immunohistochemistry

Serial brain sections showing CPu and SN were immunohistochemically reacted for TH to verify successful 6-OHDA lesioning and to exclude an additive effect of BoNT-A. Brains were fixed with 3.7% paraformaldehyde overnight and stained with monoclonal mouse anti-TH antibody (clone TH2, Sigma-Aldrich) following biotinylated horse anti-mouse lgG (Vector Laboratories, Burlingame, CA, USA, 1:67). For details of the procedure see [69].

### 4.4. Behavioral Testing

The degree of dopaminergic cell loss and the positive motor effect of BoNT-A was evaluated using apomorphine- and amphetamine-induced rotational behavior. Testing was performed in a rotometer [41] four weeks after 6-OHDA or sham-6-OHDA injection and again two weeks after BoNT-A or sham-BoNT-A injection (Figure 6). Drugs were solved in 0.9% NaCl and injected i.p. (apomorphine: 0.25 mg/kg, amphetamine: 2.5 mg/kg). Following apomorphine injection and a waiting time of 5 min to ensure cerebral uptake, rotations were monitored for 40 min. Rotational behavior induced by amphetamine application was analyzed after a waiting time of 15 min throughout a period of 60 min. 

### 4.5. Radioligand Preparation and PET/CT Imaging

Synthesis of [^18^F]fallypride ([^18^F](*S*)-*N*-((1-allylpyrrolidin-2-yl)methyl-5-(3-fluoropropyl)-2,3-dimethoxybenzamide) and semi-preparative HPLC for purification was conducted according to the protocol of [70], followed by an extensive quality control. D_2_/D_3_R availability was analyzed by dynamic [^18^F]fallypride PET/CT imaging over 90 min, each animal was measured one, three, and six months post-BoNT-A or -sham-BoNT-A injection. Anesthesia was initially administered with 5% isoflurane (AbbVie, North Chicago, IL, USA) vaporized in oxygen gas and maintained during scanning time with 1.5–3%. Body temperature was held constant at 38 °C via a heating pad and respiration rate of the animals was monitored throughout the PET/CT measurement. Each rat was placed in head-prone position centered in the field of view of a commercially available preclinical PET/CT scanner (Inveon^®^, Siemens Healthcare, Knoxville, TN, USA); performance evaluation of the system was described by [71,72]. [^18^F]fallypride was injected as a bolus over 1 min via a microcatheter into the lateral tail vein in a mean dose of 23.44 ± 1.75/24.06 ± 1.76/22.81 ± 2.03 MBq (sham-6-OHDA + sham-BoNT-A), PET/CT 1-3), 24.52 ± 2.48, 23.84 ± 2.28, 23.88 ± 2.46 MBq (6-OHDA + Sham-BoNT-A, PET/CT 1–3) and 22.99 ± 2.50, 22.55 ± 3.93, 21.61 ± 2.27 MBq (6-OHDA + BoNT-A, PET/CT 1–3). The acquisition of dynamic PET as list mode data set was started immediately with the injection. PET studies were reconstructed as series 3D PET images of multiple frames with various time durations (6 × 10 s, 8 × 30 s, 5 × 300 s, 5 × 1800 s) with a voxel size of 0.86 mm × 0.86 mm × 0.79 mm using a 2D-ordered subsets expectation maximization algorithm (four iterations, six subsets). Attenuation correction was performed on the basis of whole body CT scan and PET studies were also corrected for random coincidences, dead time, scatter and radioactive decay.

### 4.6. MRI Imaging

MRI was performed on anesthetized rats (1.5–3% isoflurane in oxygen) at least 10 days after the last PET/CT examination and about eight months post-6-OHDA lesion. MRI of the rats was conducted using a 7 Tesla small animal MRI scanner (BioSpec 70/30 AVANCE III, 7.0 T, 440 mT/m gradient strength, Paravision software v6.01., Bruker BioSpin MRI GmbH, Ettlingen, Germany) with a 1 H transmit resonator (inner diameter: 86 mm; vendor type-nr.: T12053V3, Bruker, Ettlingen, Germany) and a receive-only surface coil array (2 × 2 array rat brain coil; vendor type-nr.: T11483V3, Bruker) positioned on the head of the rats. The imaging protocol included 3D isotropic T1w FLASH imaging sequences with transversal slice orientation, 8/45 ms TE/TR, 35 mm × 35 mm × 16 mm FOV, 200 µm × 200 µm × 200 µm resolution, 175 pixel × 175 pixel × 80 pixel matrix size, 20° flip angle, 12:36 min:sec acquisition time, one average and fat suppression.

### 4.7. Image Analysis

**Figure 7:**
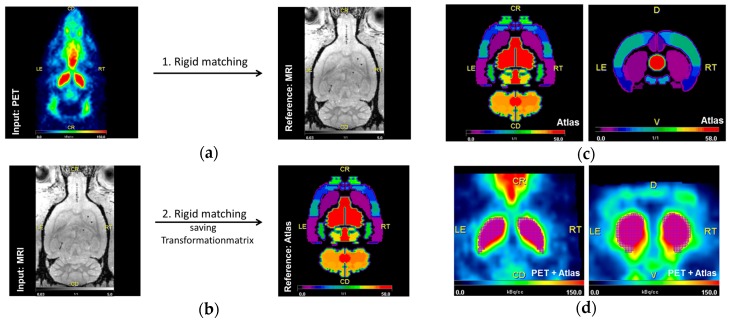
(**a**,**b**) Workflow of the transformation process using rigid matching. In a first step PET (input) was transformed to the matrix of the animal-specific MRI (reference) (**a**). Secondly, the animal-specific MRI (input) was transformed to the matrix of the Schiffer atlas and the transformation matrix was saved and finally applied to PET data (**b**). (**c**,**d**) Example of the delineation of the left and right striatum (purple grid) and the cerebellum (orange grid) in PET (**d**) with published Schiffer atlas (**c**) in a control animal (sham-6-OHDA + sham-BoNT-A).

For qualitative and quantitative analysis PMOD v3.7 (PMOD Technologies LLC, Zurich, Switzerland) was used. Qualitative assessment was conducted with parametric maps of the spatial BP_nd_ distribution by pixel-wised calculation (extracting signals from individual pixels). For determination of BP_nd_, delineation of the target region (left and right striatum) as well as the reference region (cerebellum) was conducted using an implemented MRI-based rat brain atlas [73]. Therefore, PET data were first transformed to the standard matrix of the animal-specific MRI (3D isotropic T1w FLASH). Two rats did not receive a MRI scan, for transformation a representative MRI of the same experimental group was used. Two animals died during the study course and four PET/CT scanning time points were canceled. Six PET/CT scans were excluded from analysis due to incorrect injection of the radioligand and one due to development of a brain tumor (Table 1). Animal-specific MRI datasets were then transformed to Schiffer matrix and the respective transformation matrix was saved. In a final step, PET data were transformed into a Schiffer matrix using the saved transformation matrix to guarantee maximal resolution of PET data (Figure 7a,b). All transformations were performed using ridged matching method implemented in PMOD. VOIs for target and reference region were then defined with the Schiffer atlas [73] (Figure 7c,d) and time–activity curves (TAC) were extracted from dynamic PET data. For kinetic analysis the model-driven SRTM2 [54] was applied and BP_nd_ was estimated, being defined as the ratio of receptor density (B_max_) multiplied by the radioligand affinity [74]. We assumed that receptor affinity was not changed in 6-OHDA-lesion rats compared to controls as BP of [^18^F]fallypride is resistant to DA depletion [75,76,77]. 

### 4.8. Statistical Assessment

Statistical significance was examined using IBM SPSS Statistics software version 22. To test for the Gaussian distribution of all reported data, a Kolmogorov–Smirnov test was performed followed by a univariate general linear model. Between-subjects post hoc ANOVA analysis of variance was conducted with D_2_/D_3_R availability of the CPu (left/right) as the dependent variable and the experimental group as covariate. Following, Bonferroni correction with the factor group for each of the analyzed time points was performed (df = 5; (F = 4.598 one month post-BoNT-A or -sham-BoNT-A; F = 3.051 three months post-BoNT-A or -sham-BoNT-A, F = 2.584 six months post-BoNT-A or -sham-BoNT-A)). Statistical significance of apomorphine- and amphetamine-induced behavior was analyzed by the unpaired student’s *t*-test. To analyze correlations of D_2_/D_3_R availability and rotational behavior in the apomorphine-induced rotation test linear regression followed by a two-sided Pearson correlation test was implemented. A *p*-value below 0.05 was considered to indicate significance.

## 5. Conclusions

We here provide a longitudinal study on changes of D_2_/D_3_R availability in the 6-OHDA-induced hemi-PD rat model. We found an increase in D_2_/D_3_R availability of 23% up to six months post-lesion, which was significantly reduced after striatal injection of BoNT-A. Interestingly, this decrease of pathological D_2_/D_3_R imbalance by intrastriatal BoNT-A injection significantly correlated with behavior in the apomorphine rotation test. Altogether, our results emphasize the therapeutical capability of BoNT-A in hemi-PD rats and provide insights in the underlying mechanisms.

## Figures and Tables

**Table 1:** Summary of all single BP_nd_ values of D_2_/D_3_R for the left and right CPu and the interhemispheric difference relative to the left hemisphere in (%) analyzed in controls (sham-6-OHDA + sham-BoNT-A), sham-injected hemi-PD rats (6-OHDA + sham-BoNT-A) and BoNT-A-injected hemi-PD rats (6-OHDA + BoNT-A). Data are shown for all three PET/CT scans (PET/CT 1: one month post-BoNT-A or sham-BoNT-A, PET/CT 2: three months post-BoNT-A or -sham-BoNT-A, PET/CT 3: six months post-BoNT-A or -sham-BoNT-A). * indicate that no data were analyzed due to incorrect tracer injection or no data acquisition.

Group	PET/CT 1			PET/CT 2			PET/CT 3		
	BP_nd_ Left	BP_nd_ Right	(%)	BP_nd_ Left	BP_nd_ Right	(%)	BP_nd_ Left	BP_nd_ Right	(%)
sham-6-OHDA + sham-BoNT-A sham-6-OHDA + sham-BoNT-A sham-6-OHDA + sham-BoNT-A sham-6-OHDA + sham-BoNT-A sham-6-OHDA + sham-BoNT-A sham-6-OHDA + sham-BoNT-A sham-6-OHDA + sham-BoNT-A sham-6-OHDA + sham-BoNT-A sham-6-OHDA + sham-BoNT-A	5.06 2.92 4.53 4.58 4.03 * * * *	4.98 2.92 4.53 4.59 3.80 * * * *	−1.66 −0.01 −0.04 0.28 −5.71 * * * *	2.21 3.74 2.87 4.25 4.24 3.81 4.73 4.27 4.23	2.07 3.93 2.78 4.15 3.93 3.46 4.89 4.32 4.13	−6.35 5.21 −3.37 −2.28 −7.22 −9.32 3.34 1.04 −2.48	* 4.56 3.29 4.65 4.07 4.68 2.80 4.50 4.07	* 4.44 3.23 4.53 4.02 4.52 2.97 4.66 4.30	* −2.66 −1.84 −2.46 −1.36 −3.47 6.23 3.60 5.66
|Mean| ± SD	4.2 ± 0.8	4.2 ± 0.8	1.4 ± 2.5	3.8 ± 0.8	3.7 ± 0.9	2.4 ± 4.9	4.1 ± 0.7	4.1 ± 0.7	0.4 ± 4.0
6-OHDA + sham-BoNT-A 6-OHDA + sham-BoNT-A 6-OHDA + sham-BoNT-A 6-OHDA + sham-BoNT-A 6-OHDA + sham-BoNT-A 6-OHDA + sham-BoNT-A 6-OHDA + sham-BoNT-A	4.86 5.41 3.77 4.08 3.96 4.49 *	6.53 6.65 4.67 4.71 4.79 5.54 *	34.37 22.90 23.84 15.53 21.02 23.50 *	6.11 * 3.35 4.44 * 4.74 3.53	7.67 * 4.54 4.88 * 5.64 4.71	25.53 * 35.56 10.04 * 18.80 33.46	4.11 4.66 5.45 5.21 * 4.33 *	4.59 5.54 7.26 5.83 * 5.37 *	11.53 19.07 33.13 11.98 * 23.95 *
|Mean| ± SD	4.4 ± 0.6	5.5 ± 0.9	23.5 ± 6.1	4.4 ± 1.1	5.5 ± 1.3	24.7 ± 14.8	4.8 ± 1.0	5.7 ± 1.0	19.9 ± 11.4
6-OHDA + BoNT-A 6-OHDA + BoNT-A 6-OHDA + BoNT-A 6-OHDA + BoNT-A 6-OHDA + BoNT-A 6-OHDA + BoNT-A 6-OHDA + BoNT-A 6-OHDA + BoNT-A 6-OHDA + BoNT-A 6-OHDA + BoNT-A	4.58 4.54 5.07 5.09 4.85 4.37 4.59 3.88 5.30 5.10	5.33 4.90 5.54 5.42 5.43 5.15 5.12 4.28 6.22 5.59	16.19 7.94 9.23 6.39 11.80 17.86 11.55 10.26 17.22 9.74	* 5.47 4.41 2.43 * * 3.88 4.33 4.94 5.32	* 6.10 5.15 2.79 * * 4.06 5.13 5.26 6.41	* 11.59 16.68 14.93 * * 4.77 18.59 6.49 20.38	3.30 * 2.63 4.74 5.88 4.86 3.63 5.00 4.58 5.01	3.92 * 3.13 5.25 6.38 6.03 3.87 5.93 5.23 5.78	18.62 * 19.11 10.77 8.56 24.05 6.45 18.53 14.22 15.38
|Mean| ± SD	4.7 ± 0.4	5.3 ± 0.5	11.8 ± 4.0	4.4 ± 1.0	5.0 ± 1.2	13.4 ± 6.0	4.4 ± 1.0	5.1 ± 1.1	15.1 ± 5.7

## Abbreviations

BSA	Bovine serum albumin
BP_nd_	non-displaceable binding potential
BoNT-A	Botulinum neurotoxin A
CPu	caudate–putamen
D_2_/D_3_R	D_2_/D_3_ receptor
DA	dopamine
hemi-PD	hemiparkinsonian
MFB	medial forebrain bundle
MRI	magnetic resonance imaging
MSN	medium spiny neuron
PD	Parkinson’s disease
PET	positron emission tomography
PBS	phosphate-buffered saline
PVE	partial volume effect
SNAP25	synaptosomal-associated protein of 25-kDa
SN(pc)	substantia nigra (pars compacta)
SRTM2	simplified reference tissue model 2
TAC	time-activity curve
TH	tyrosine hydroxylase
VOI	voxels of interest
6-OHDA	6-hydroxdopamine
